# A Case Report of Autoimmune Glial Fibrillary Acidic Protein Astrocytopathy Diagnosed After Long Term Diagnosis of Chronic Lymphocytic Inflammation With Pontine Perivascular Enhancement Responsive to Steroids

**DOI:** 10.3389/fneur.2020.598650

**Published:** 2020-11-17

**Authors:** He-Xiang Yin, Yan Zhou, Yan Xu, Ming-Li Li, Zhe Zhang, Li-Xin Zhou, Yao Zhang, Yi-Cheng Zhu, Bin Peng, Li-Ying Cui

**Affiliations:** ^1^Department of Neurology, Peking Union Medical College Hospital, Peking Union Medical College, Chinese Academy of Medical Sciences, Beijing, China; ^2^Department of Radiology, Peking Union Medical College Hospital, Peking Union Medical College, Chinese Academy of Medical Sciences, Beijing, China; ^3^Neurosciences Center, Chinese Academy of Medical Sciences, Beijing, China

**Keywords:** chronic lymphocytic inflammation with pontine perivascular enhancement responsive to steroids, differential diagnosis, outcome, case report, autoimmune glial fibrillary acidic protein astrocytopathy

## Abstract

Cases of autoimmune glial fibrillary acidic protein (GFAP) astrocytopathy who were initially diagnosed with chronic lymphocytic inflammation with pontine perivascular enhancement responsive to steroids (CLIPPERS) were rarely reported. Herein, we reported a 31-year-old woman who presented with 7 years of recurrent headache. Her clinical history, symptoms, brain MRI enhancement features, and response to treatment during each attack were reviewed. Her brain MRI 7 years ago demonstrated characteristic pepper-like enhancement of pontine and cerebellum and her symptoms resolved completely after taking a high-dose of steroids. She was suspected with the diagnosis of CLIPPERS, and she experienced five relapses once the oral steroid was tapered below 20 mg/day. During her last relapse, she experienced fever and psychosis, and GFAPα-antibodies were detected in her serum and cerebrospinal fluid by antigen-transfected HEK293 cell-based assay (indirect immunofluorescence assay). She obtained relief again after steroid therapy, and her diagnosis converted to autoimmune GFAP astrocytopathy. Autoimmune GFAP astrocytopathy may mimic CLIPPERS, both clinically and radiologically. Long-term follow-up is essential for necessary diagnosis revision at each new attack in patients with a diagnosis of CLIPPERS.

## Introduction

Since first described in 2010 (1), cases of chronic lymphocytic inflammation with pontine perivascular enhancement responsive to steroids (CLIPPERS) were reported around the world. Lacking specific diagnosis markers, it is more like a tricky clinical syndrome with more than one kind of mimics such as primary angiitis of the central nervous system (CNS), primary CNS lymphoma, CNS demyelinating disease, and CNS lymphomatoid granulomatosis. As another inflammatory CNS disorder similar to CLIPPERS, autoimmune glial fibrillary acidic protein (GFAP) astrocytopathy was first proposed in 2016 by Lennon et al. (2). Cases of CLIPPERS reported before 2016 might actually have an autoimmune GFAP astrocytopathy, and the novel antibody test makes it possible to differentiate these two rare diseases. Herein, we describe a rare case in which GFAPα-antibody was detected in a patient diagnosed with probable CLIPPERS for the long-term to call for awareness of this potential rare but important clinical condition.

## Case Presentation

**Figure 1:**
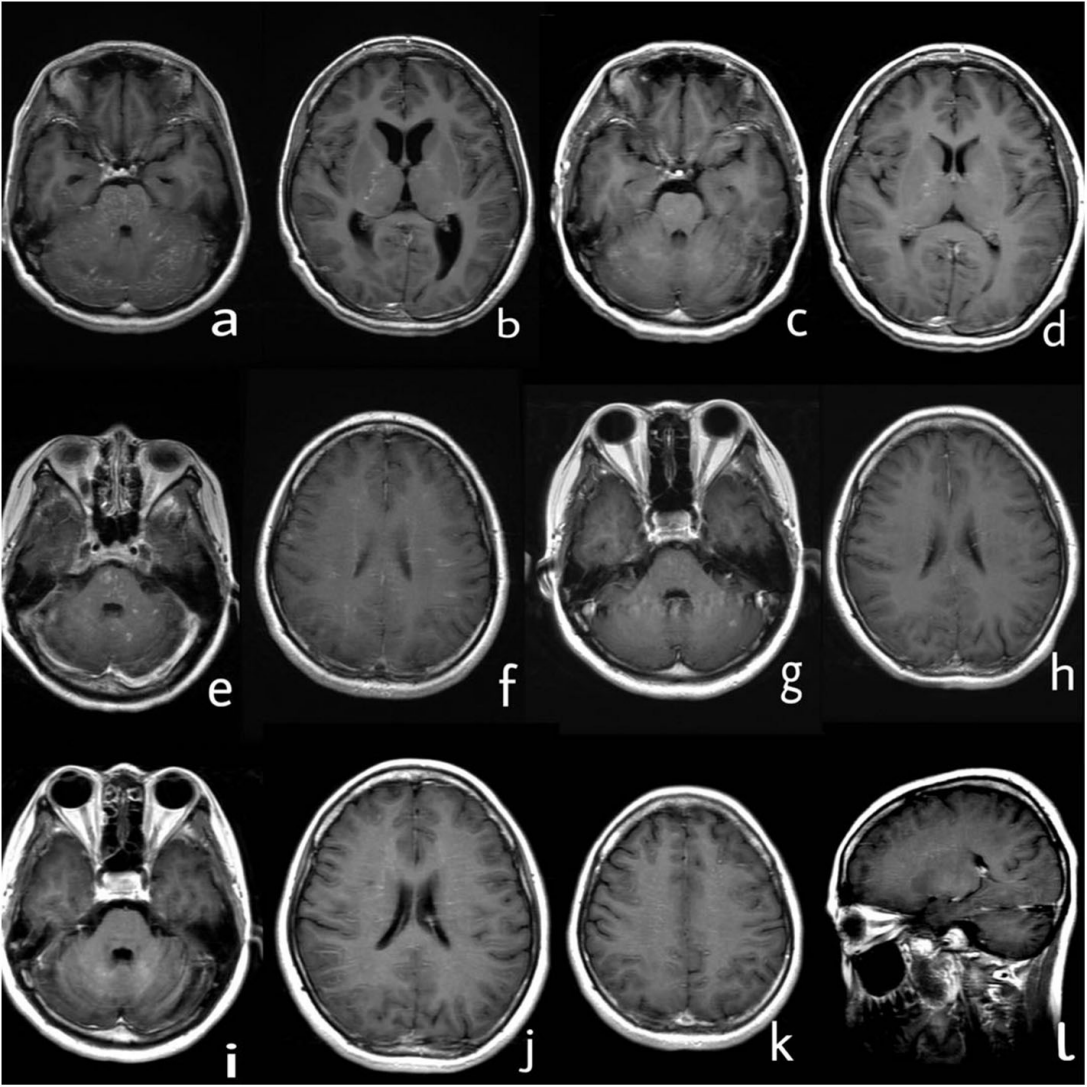
Series of brain MRI with contrast. **(a,b)** In March 2012, curvilinear and punctate enhancements of pontine, cerebellum, and basal ganglion were demonstrated. **(c,d)** In June 2012, enhancement of pontine, cerebellum, and basal ganglion improved after treatment with steroids. **(e,f)** In June 2015, novel dot-like and liner enhancement in pontine, semiovale center, and juxtacortical area were shown. **(g,h)** In September 2015, enhancement in pontine, semiovale center, and juxtacortical area improved again after treatment with steroids. **(i–l)** In September 2019, novel dot-like and liner enhancement in both hemispheres of the cerebellum, semiovale center, basal ganglion, and thalamus were demonstrated.

A 31-year-old woman presented with 7 years of recurrent head discomfort and 2 days of fever with nausea, vomiting, and intermittent symptom of psychosis. The patient's symptoms started in February 2012. She developed headache, nausea, and vomiting, then accompanied by numbness of bilateral forehead and paresthesia of her face and back. Her initial brain magnetic resonance imaging (MRI) after 1 month revealed multiple areas of curvilinear and punctate enhancement of pontine, cerebellum, and basal ganglion (Figures 1a,b). After possible infection and tumor were ruled out, she was prescribed with 1 g of intravenous methylprednisolone (IVMP) per day for five consecutive days, 500 mg of IVMP per day for the next 3 days. Then, 60 mg of oral prednisone was administrated and tapered. She had rapid clinical and radiological improvements (Figures 1c,d). Even without neuropathological evidence, the diagnosis of probable CLIPPERS was determined based on the imaging characteristic and treatment response. During the regular follow-up at the outpatient clinic in the past 7 years, the patient's symptom and/or brain MRI enhancement deteriorated every time the oral prednisone tapered near the dose of 20 mg per day, although immunosuppressive agent such as azathioprine was tried (Table 1, Figures 1e,f). However, she obtained relief soon after high-dose IVMP or oral prednisone was added on (Figures 1g,h).

**Table 1:** Patient's clinical data of each attack or relapse.

**Attack**	**1**	**2**	**3**	**4**	**5**	**6**	**7**
Time from disease onset	Onset	+6 months	+24 months	+39 months	+45 months	+51 months	+87 months
Symptoms	Headache, nausea and vomiting, numbness of bilateral forehead and paresthesias of the face and back	Stable	Dizziness,vertigo, nausea, vomiting, numbness of head	Leg numbness after a long walk	Paroxysmal tension of the head	Stable	Headache, fever, nausea, vomiting and intermittent symptom of psychosis
CSF studies							Day 2; Day 7
WBC (cell/mm^3^)	2	3	NA	NA	2	NA	10; 6
Protein (mg/dl)	40	38	NA	NA	50	NA	65; 55
Cytology	Lymphocytosis	Slight lymphocytosis	NA	NA	Normal	NA	Normal
OCB	Negative	Negative	NA	NA	±	NA	Negative
Anti-GFAP Ab serum titer; CSF titer	NA	NA	NA	NA	NA	NA	1:320; 1:32
Brain MRI enhancement lesions	Pontine, cerebellum, and basal ganglion (Figures 1a,b)	Right internal capsule, left thalamus, pontine, cerebellum	Pontine, cerebellum	Pontine, left basal ganglion, semiovale center and juxtacortical area of the frontal and parietal lobe (Figures 1e,f)	Pontine, cerebellum and juxtacortical area of the bilateral cerebral lobe	Pontine, cerebellum semiovale center and subcortical area of the bilateral cerebral lobe	Cerebellum, semiovale center, basal ganglion, and thalamus (Figures 1i–l)
Treatment before attack	None	Prednisone 12.5 mg/day	Prednisone 12.5 mg/day	Prednisone 10 mg/day AZA 50 mg/day	Prednisone 15 mg/day AZA 100 mg/day	Prednisone 20 mg/day	None (stop treatment by herself)
Treatment after attack	High-dose IVMP	Prednisone 40 mg/day	Prednisone 50 mg/day AZA 50 mg/day	Prednisone 50 mg/day AZA 100 mg/day	High-dose IVMP	Prednisone 40 mg/day	High-dose IVMP
Clinical response to therapy	Complete	Complete	Complete	Complete	Complete	Complete	Complete
Radiological response to therapy	Nearly complete	Complete	Complete	Nearly complete	Complete	Complete	Nearly complete

*IVMP, intravenous methylprednisolone; AZA, azathioprine; WBC, white blood cell; OCB, oligoclonal band; NA, not available*.

The patient discontinued oral prednisone by herself in June 2019 (7 years from the disease onset), and her headache attacked again. She had another head MRI examination in September 2019, and it showed novel dot-like and liner enhancement in both hemispheres of the cerebellum, semiovale center, basal ganglion, and thalamus (Figures 1i–l). She refused repeated treatment of steroids then. Her symptoms deteriorated in November 2019, and she had a fever with nausea, vomiting, and intermittent symptoms of psychosis, which brought her to the hospital again.

**Figure 2:**
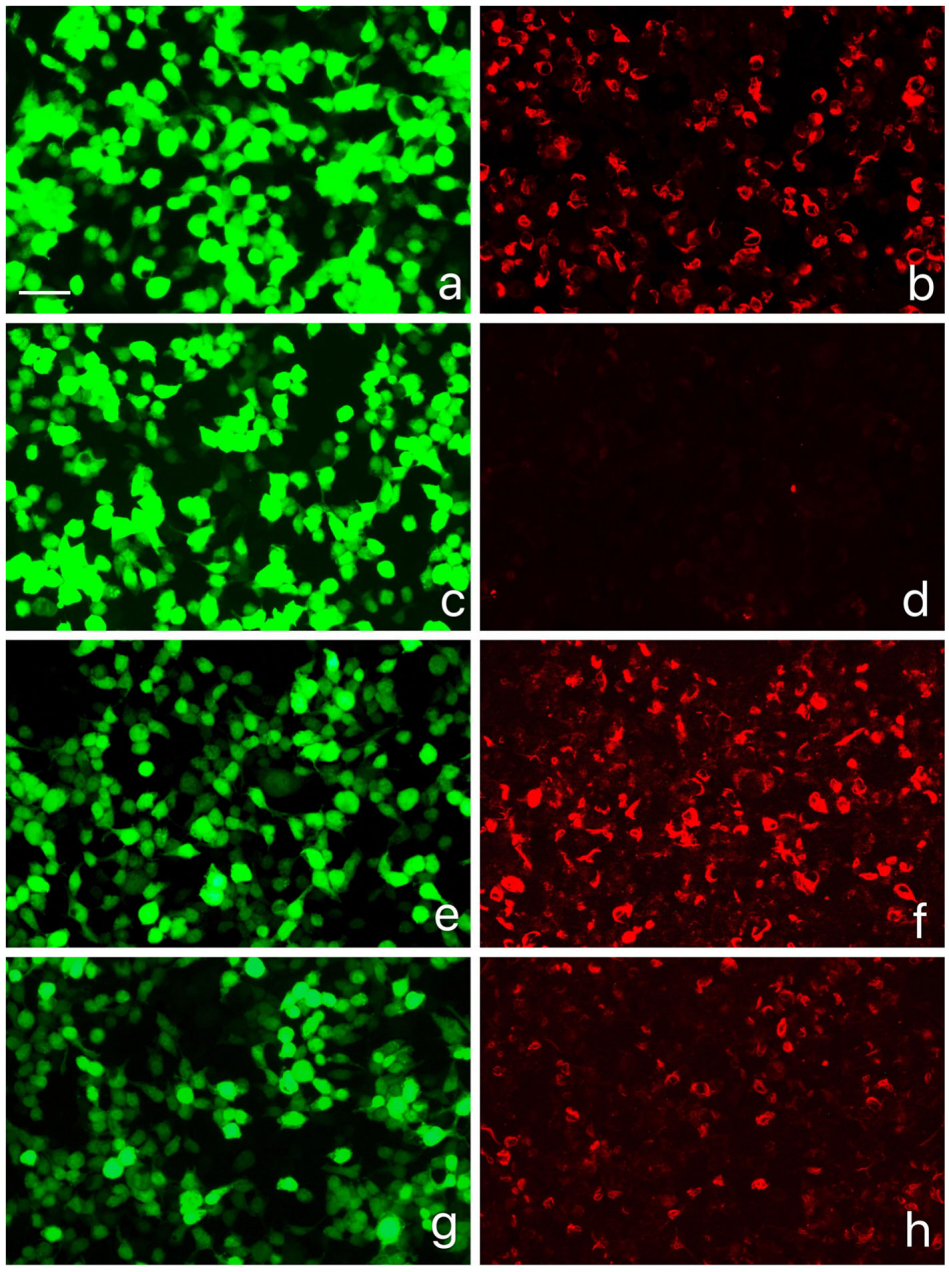
GFAPα-IgG test results by GFAP-transfected HEK293 cell-based immunofluorescence assay. **(a,c,e,g)** HEK293 cells expressing green fluorescent protein (GFP)-tagged GFAP (green). **(b,d,f,h)** HEK293 cells immunostained with human IgG (red if positive): **(b)** Positive control with human anti-GFAPα IgG, **(d)** negative with healthy control, **(f)** positive result of serum (titer at 1:320), **(h)** positive result of cerebrospinal fluid (CSF) (titer at 1:32). Scale bar = 50 *um*.

The physical examination showed delirium, suspected stiff neck, and positive result of both palmomental reflex and left Hoffmann sign, whereas the remaining general and neurological examination results were generally normal. Laboratory test results, including C-reactive protein, serum lactate dehydrogenase, antinuclear antibodies, myelin oligodendrocyte glycoprotein immunoglobulin G, antineuronal, and antiganglioside antibodies, were normal. The cerebrospinal fluid (CSF) analysis was unremarkable. Further antibody analysis by antigen-transfected HEK293 cell-based assay (Supplementary Material 1) showed positive GFAPα antibodies with serum titer at 1:320 and CSF titer at 1:32 (Figure 2). She was prescribed with IVMP, 1 g daily for five consecutive days, and she was discharged with oral prednisone taper and mycophenolate mofetil for relapse prevention when she returned normal again 1 week later. Her diagnosis was converted from CLIPPERS to autoimmune GFAP astrocytopathy, and she is still under follow-up.

## Discussion

In retrospect, our patient has several atypical features to fulfill the revised criteria for CLIPPERS (3). She did not always have classical manifestations of subacute pontocerebellar dysfunction, such as gait ataxia and diplopia, during each attack. Notably, at the recent attack, she experienced symptoms of meningitis, a classical type of GFAP astrocytopathy, which made the diagnosis of CLIPPERS less likely. On the other hand, the enhancement characteristics of MRI changes over time. The enhancement lesions dominated in the infratentorial compartment, such as the cerebellum and pons, and appeared as homogenous enhancing nodules at the onset. In contrast, during later attacks, the enhancements were mainly distributed in the supratentorial compartment, such as the semiovale center and juxtacortical area, and presented as linear perivascular radial characteristics. In the largest reported case series of GFAP astrocytopathy (4), relapses occurred in approximately 20% of patients during steroid taper, making it hard to differentiate GFAP astrocytopathy from CLIPPERS based on treatment response. However, it is proposed that relapse with atypical symptoms or radiological features should be a “red flag” to the diagnosis of CLIPPERS (5), and the patient in our case showed unusual relapse, which made the diagnosis revised. It is a pity that the blood or CSF sample of the patient's previous attacks was not available for antibody tests; therefore, it is difficult to conclude that the patient had GFAP astrocytopathy from the beginning. After all, diagnosis of CLIPPERS seems to be the most optimal explanation during the patient's first attack in 2012.

Classical neuropathology of CLIPPERS revealed dense lymphocytic inflammation (T cells predominance) with perivascular predominance and parenchymal diffuse infiltration and absence of myelin loss or focal secondary myelin loss (5); there is no conclusive etiology and pathogenesis for it yet. Similarly, limited cases with the neuropathology of GFAP astrocytopathy showed extensive inflammation (infiltration of lymphocytes, monocytes, and neutrophils) around the vessels (6), which was consistent with linear perivascular radial gadolinium enhancement characteristics, whereas the loss of astrocytes, neuron loss, and demyelination were not always observed. However, B cells and plasma cells were also observed in the lesion area Virchow–Robin spaces, which is different from CLIPPERS. It is speculated that certain triggers such as infection in some cases of CLIPPERS patients prompt that the perivascular T lymphocytes interact with B cells and other inflammatory components of the immune system (microglia, macrophages, and antibody secretion by plasma cells), contributing to the pathogenesis of GFAP neurological autoimmunity. Whether GFAP astrocytopathy is one of the clinical outcomes for cases of CLIPPERS still remained to been explored.

## Conclusions

Autoimmune GFAP astrocytopathy may mimic CLIPPERS, both clinically and radiologically. Even in patients with a diagnosis of CLIPPERS, long-term follow-up is essential for necessary diagnosis revision at each new attack, and the GFAP-α antibody test was suggested for differential diagnosis, especially when the patient presents with linear perivascular radial gadolinium enhancement.

## Data Availability Statement

The original contributions presented in the study are included in the article/Supplementary Material, further inquiries can be directed to the corresponding author/s.

## Ethics Statement

Written informed consent was obtained from the individual(s) for the publication of any potentially identifiable images or data included in this article.

## Author Contributions

H-XY reviewed the literature and drafted the manuscript. YZho took care of the patient and conceptualized the study. YX designed the study, oversaw data acquisition, and supervised the initial drafting. M-LL provided MRI diagnosis advice. ZZ, L-XZ, and YZha took care of the patient and acquired the clinical data. Y-CZ, BP, and L-YC critically revised the manuscript. All authors contributed to the article and approved the submitted version.

## Conflict of Interest

The authors declare that the research was conducted in the absence of any commercial or financial relationships that could be construed as a potential conflict of interest.
