# Supplementary material for: A Case Report of Autoimmune Glial Fibrillary Acidic Protein Astrocytopathy Diagnosed After Long Term Diagnosis of Chronic Lymphocytic Inflammation With Pontine Perivascular Enhancement Responsive to Steroids
Source: Front Neurol. 2020 Nov 17;11:598650. doi: 10.3389/fneur.2020.598650 (PMC7705065; doi:10.3389/fneur.2020.598650)
Supplement: Supplementary file 1 [file Data_Sheet_1.DOCX]

**Cell-based assay**

Stable clones of Human embryonic kidney(HEK)-293 cells were co-transfected with pcDNA-GFAPα and pEGFP-N1 using Lipofectamine 2000 (Invitrogen, transcription efficiency was 60-80%).Thirty-six hours after transfection, the HEK-293 cells were fixed with 4% paraformaldehyde for 20 minutes and permeabilized with 0.1% Triton X-100 in phosphate-buffered saline (PBS) for 20 min. Cells were incubated with patient’s serum or cerebrospinal fluid(CSF) for 2 hours and then immunolabeled with an AlexaFluor 546 secondary antibody against human IgG (1:1000; Thermo Scientific) for 1 hour at room temperature. Images were acquired using a Zeiss Axiovert A1 fluorescence microscope.
