# Supplementary material for: A Case Report of Autoimmune Glial Fibrillary Acidic Protein Astrocytopathy Diagnosed After Long Term Diagnosis of Chronic Lymphocytic Inflammation With Pontine Perivascular Enhancement Responsive to Steroids
Source: Front Neurol. 2020 Nov 17;11:598650. doi: 10.3389/fneur.2020.598650 (PMC7705065; doi:10.3389/fneur.2020.598650)
Supplement: Supplementary file 2 [file Data_Sheet_2.PDF]

CARE Checklist (2013) of information to include when writing a case report

(cc) BY-NC-ND

✓

Topic	Item	Checklist item description	Reported on Page
**Title**	1	The diagnosis or intervention of primary focus followed by the words "case report"	Page 1, Title
**Key Words**	2	2 to 5 key words that identify diagnoses or interventions in this case report, including "case report"	Page 2, Keywords
**Abstract (no references)**	3a	Introduction: What is unique about this case and what does it add to the scientific literature?	Page 2, Abstract
	3b	Main symptoms and/or important clinical findings	Page 2, Abstract
	3c	The main diagnoses, therapeutics interventions, and outcomes	Page 2, Abstract
	3d	Conclusion—What are the main "take-away" lesson(s) from this case?	Page 2, Abstract
**Introduction**	4	One or two paragraphs summarizing why this case is unique (may include references)	Page 2, Introduction
**Patient Information**	5a	De-identified patient specific information	Page 3-4, Case report
	5b	Primary concerns and symptoms of the patient	Page 3-4, Case report
	5c	Medical, family, and psychosocial history including relevant genetic information.	Page 3-4, Case report
	5d	Relevant past interventions with outcomes	Page 3-4, Case report
**Clinical Findings**	6	Describe the relevant physical examination (PE) and other significant clinical findings.	Page 3-4, Case report
**Timeline**	7	Important information from the patient's history organized as a timeline	Page 9-10, Table 1
**Diagnostic Assessment**	8a	Diagnostic methods (such as PE, laboratory testing, imaging, surveys).	Page 3-4, Case report
	8b	Diagnostic challenges (such as access, financial, or cultural)	Page 3-4, Case report
	8c	Diagnostic reasoning including other diagnoses considered	Page 3-4, Case report
	8d	Prognostic characteristics (such as staging in oncology) where applicable	**N/A**
**Therapeutic Intervention**	9a	Types of intervention (such as pharmacologic, surgical, preventive, self-care)	Page 3-4, Case report
	9b	Administration of intervention (such as dosage, strength, duration)	Page 3-4, Case report

Section	Item	Description	Page
	9c	Changes in intervention (with rationale)	Page 3-4, Case report
**Follow-up and Outcomes**	10a	Clinician and patient-assessed outcomes (when appropriate)	Page 3-4, Case report
	10b	Important follow-up diagnostic and other test results	Page 3-4, Case report
	10c	Intervention adherence and tolerability (How was this assessed?)	N/A
	10d	Adverse and unanticipated events	N/A
**Discussion**	11a	Discussion of the strengths and limitations in your approach to this case	Page 4-5, discussion
	11b	Discussion of the relevant medical literature.	Page 4-5, discussion
	11c	The rationale for conclusions (including assessment of possible causes)	Page 4-5, discussion
	11d	The primary "take-away" lessons of this case report	Page 4-5, discussion
**Patient Perspective**	12	When appropriate the patient should share their perspective on the treatments they received	N/A
**Informed Consent**	13	Did the patient give informed consent? Please provide if requested	Yes ☐√ No ☐
